# Supplementary material for: From experimental clues to theoretical modeling: Evolution associated with the membrane-takeover at an early stage of life
Source: PLoS Comput Biol. 2025 Jun 13;21(6):e1012763. doi: 10.1371/journal.pcbi.1012763 (PMC12201655; doi:10.1371/journal.pcbi.1012763)
Supplement: S1 Text — (PDF) [file pcbi.1012763.s001.pdf]

Supporting Information for:

From experimental clues to theoretical modeling: Evolution associated with the membrane-takeover at an early stage of life

Wentao Ma [1]*, Chunwu Yu [2]

1. Hubei Key Laboratory of Cell Homeostasis, College of Life Sciences, Wuhan University, Wuhan, China

2. College of Computer Sciences, Wuhan University, Wuhan, China

* Correspondence to:

Wentao Ma

College of Life Sciences,

Wuhan University,

Wuhan,

P. R. China

Email: mwt@whu.edu.cn

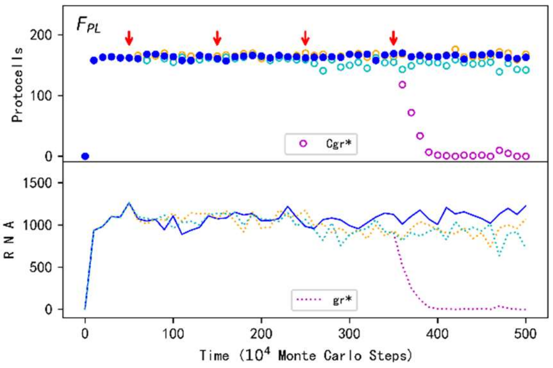

Fig A. The influence of F_{PL} and P_{PLM} on the spread of the protocells containing GR. The situation is the same as that shown in Fig 3-F_{PL}, except that at the fourth change point of the turning-down case (cyan symbols, where F_{PL} equals to 0), instead of changing P_{FLM}, P_{PLM} is changed from its default value 1×10^{-4} to 0.002 (the legends Cgr* and gr* refer to this change).

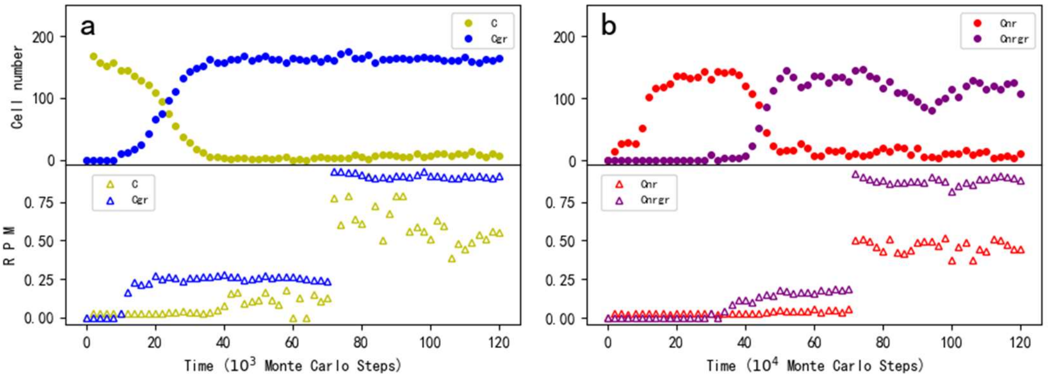

Fig B. The influence of P_{PF} on the content of membrane components. The legends are the same as those in Fig 5. (**a**) Based on the case of Fig 5a, at step 7×10^{5}, P_{PF} is changed from its default value 0.02 to a value of 0.2. (**b**) Based on the case of Fig 5b, at step 7×10^{6}, P_{PF} is changed from its default value 0.02 to a value of 0.2. With the increase of RPM for those protocells with GR (i.e., Cgr in **a** and Cnrgr in **b**), the RPM for the protocells without GR (i.e., C in **a** and Cnr in **b**) also increases, which should be attributed to phospholipids' exchange between protocells.

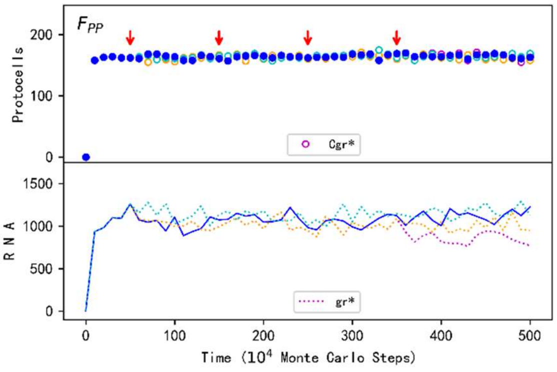

Fig C. The influence of F_{PP} and P_{PF} on the spread of the protocells containing GR. The situation is the same as that shown in Fig 3-F_{PP}, except that at the fourth change point of the turning-down case (orange symbols, where F_{PP}=2×10^4), it is P_{PF} (instead of F_{PPW}) that is changed – from its default value 0.02 to 0.2 (the legends Cgr* and gr* refer to this change).

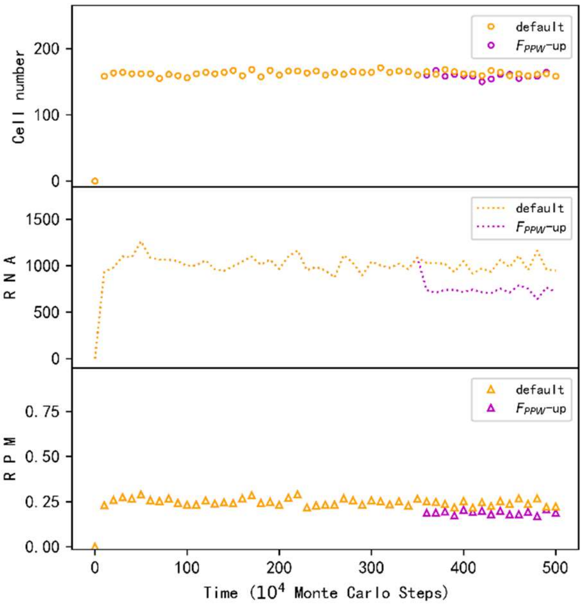

Fig D. The corresponding decline of GR protocells' phospholipid content in the membrane (RPM) with the turning up of F_{PPW}. The cases are the same as those shown in Fig 3-F_{PP} (concerning orange and purple symbols). Legends: "default" represents the case without the change of F_{PPW} (i.e. with a default value of 3), while "F_{PPW}-up" represents the case that F_{PPW} is changed from its default value to 3000 at step 3.5×10^6.

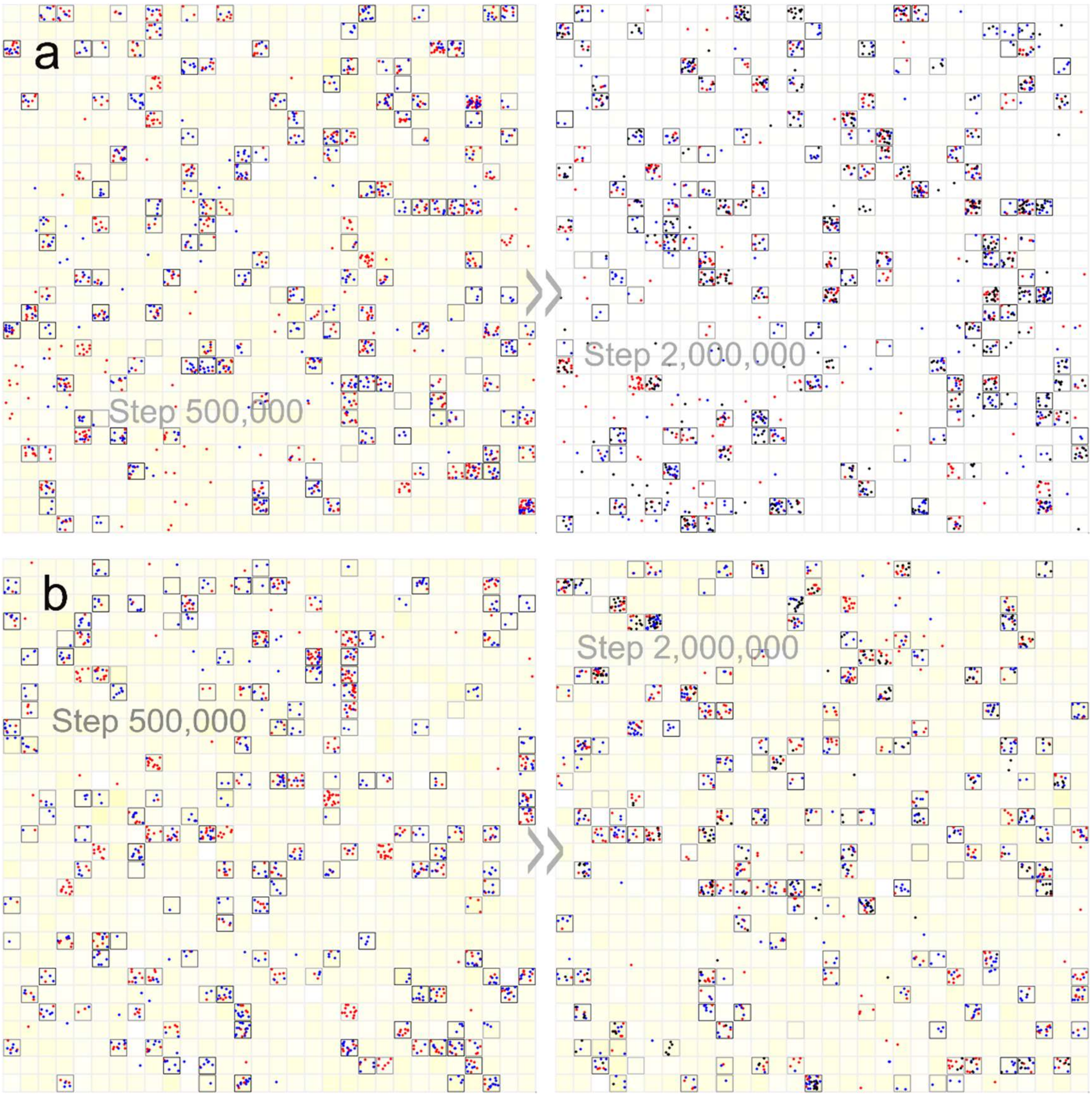

Fig E. The snapshots on spatial distribution of the cases showing the spread of new functional RNA species in NR-GR protocells due to the decreased membrane permeability. The evolutionary dynamics of the two cases are shown in Fig 6. In addition to the symbols that are explained the same way as those included in Fig 4, here black dots are introduced to denote new functional RNA species. (**a**) The black dots denote NPR molecules. The left subfigure (step 500,000) represents the stage before the spread of NPR (that is, only NR and GR exist), whereas the right subfigure (step 2,000,000) represents the stage after the spread of NPR. Notably, after the spread of NPR, its substrates, i.e. precursors of nucleotide precursors, which are represented by color-depth of the background yellow, are almost exhausted. (**b**) The black dots denote TR molecules. The left subfigure (step 500,000) represents the stage before the spread of TR, whereas the right subfigure (step 2,000,000) represents the stage after the spread of TR. The phenomenon of the precursors of nucleotide precursors' exhaustion is not observed – because the function of TR is to facilitate the across-membrane transport of nucleotide precursors rather than to exploit precursors of nucleotide precursors.

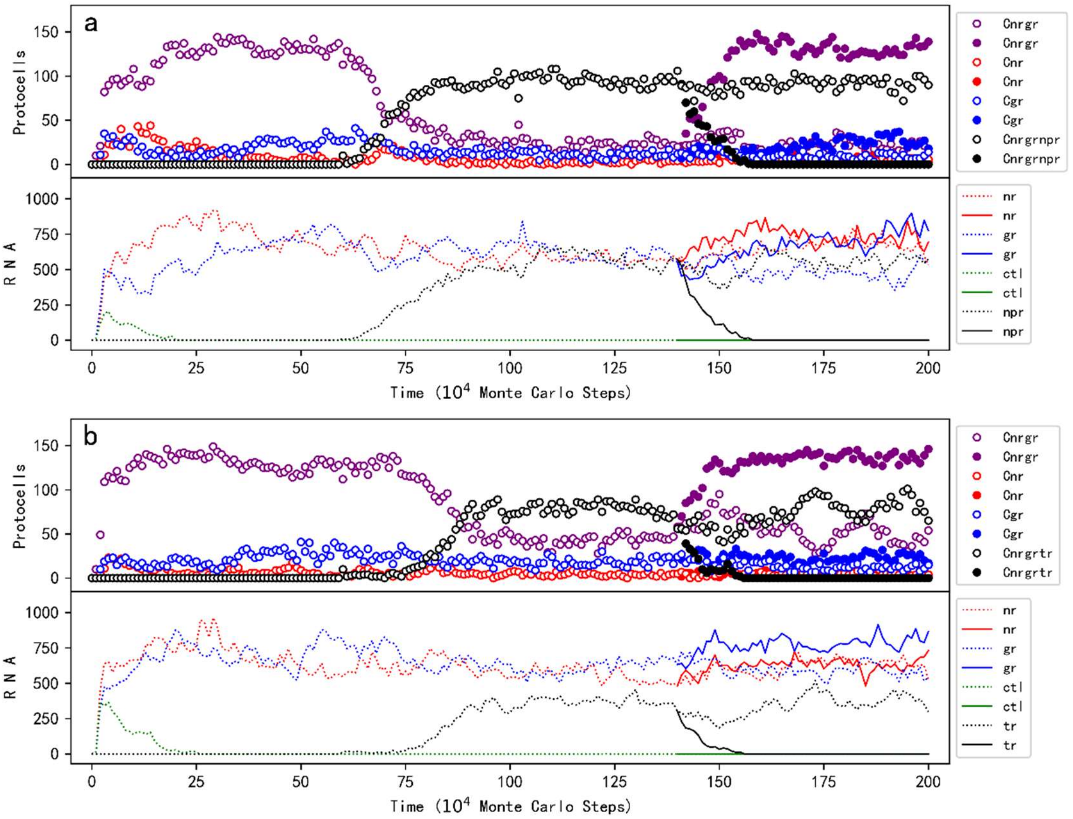

Fig F. The spread of the ribozyme exploiting more fundamental raw materials (NPR) and that of the RNA species favoring membrane transport (TR) are attributed to their function. The figure is explained the same way as Fig 6, except that the cases denoted by solid circles and solid lines in Fig 6, which represent the situation without consideration of negative influence of phospholipid content on the membrane's permeability, are not shown – here, instead, solid circles and solid lines denote the cases in which the relevant function is turned off after 1.4×10^6. (**a**) The function of NPR is turned off by setting P_{NPFR} to 0. (**b**) The function of TR is turned off by setting F_{TR} to 0.

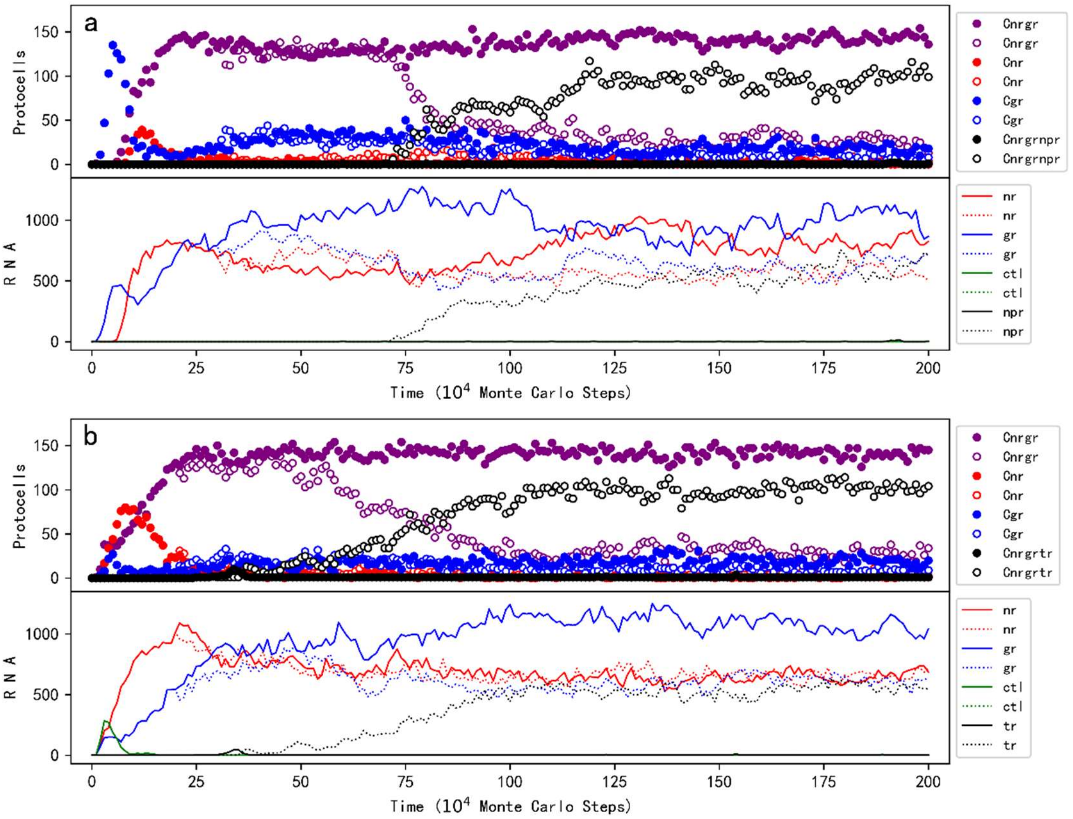

Fig G. The simulation cases modeling the emergence of NPR and TR in reality. The legends are the same as those in Fig 6. The modeling situations are the same as those in the cases of Fig 6, except the details explained in the following. At step 1×10^4, only one empty protocell is selected and inoculated with one molecule of NR, GR and the control species – note that since the raw materials in the system are initially abundant, such an inoculation (to achieve the spread of NR-GR protocells) need not be conducted repeatedly (that is, unlike the ones mentioned below). (**a**) After step 6×10^5, one molecule of NPR is inoculated into one NR-GR protocell every 1×10^5 steps. P_{BB}=5×10^{-5}, P_{NFR}=0.9 and P_{NPFR}=0.5. (**b**) For the case in which the influence of phospholipid content on membrane permeability is considered (empty circles and dotted lines), F_{PP} and F_{PPW} are turned up (to 30 and 3 respectively) at step 2×10^5. After step 2.5×10^5, one molecule of TR is inoculated into one NR-GR protocell every 1×10^4 steps. P_{BB}=5×10^{-5} and P_{NFR}=0.9.

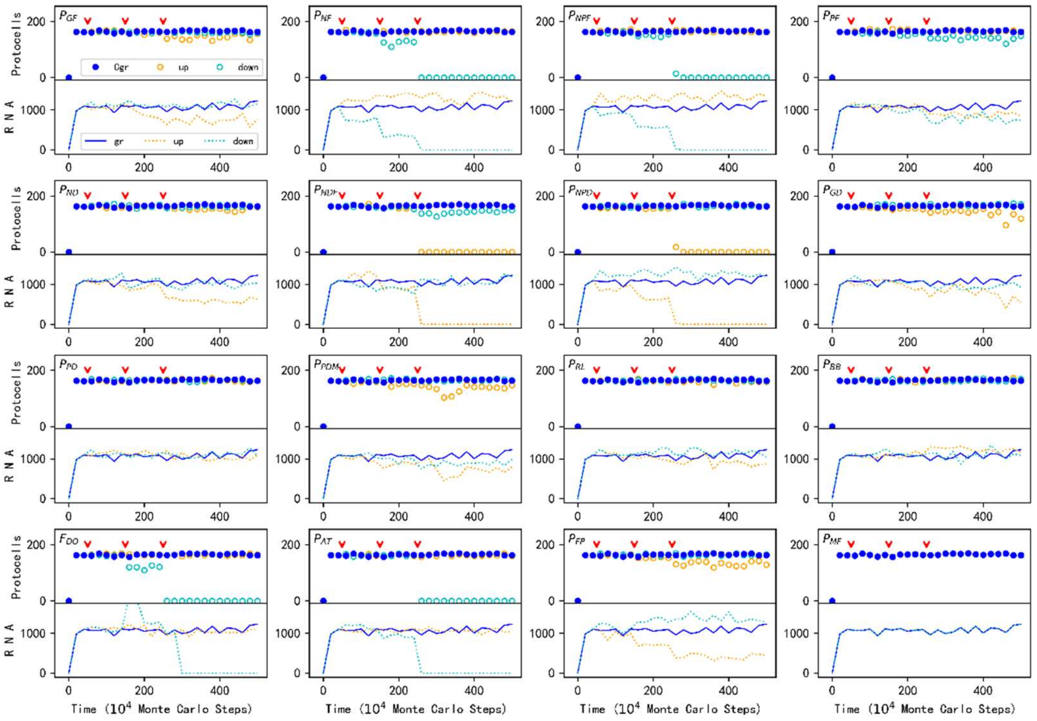

Fig H. The influence of other parameters (in addition to those shown in Fig 3) on the spread of GR protocells (part 1). The representations are the same as those in Fig 3. The values adopted at the three critical change points (red arrowheads) are listed in Table A. See Box A for a comment on the influence.

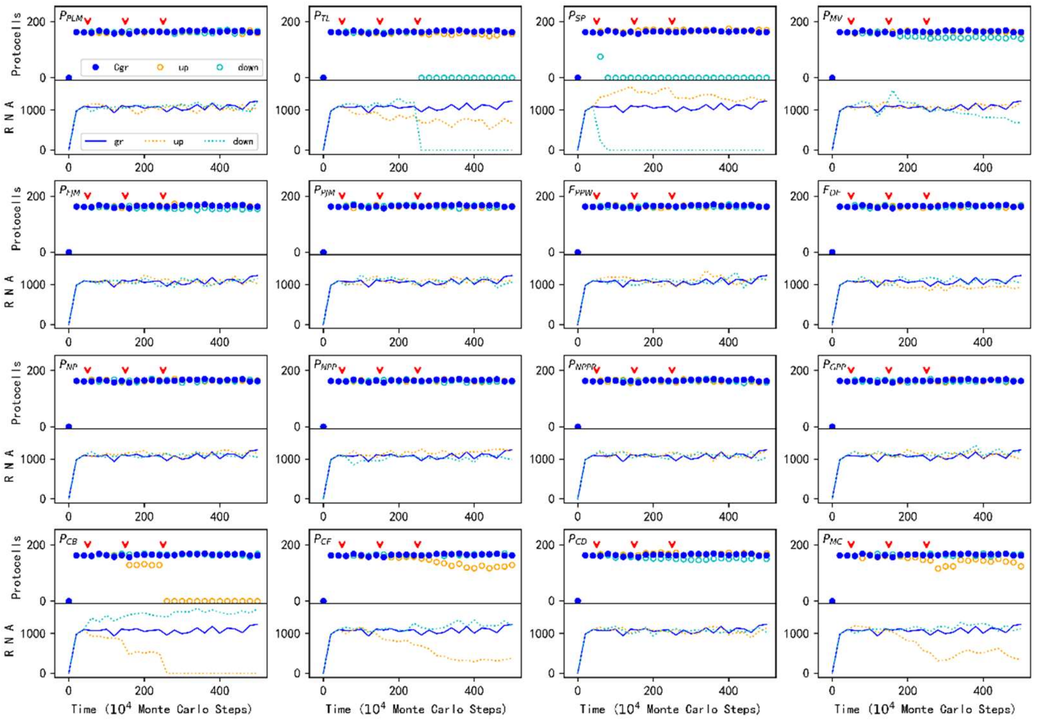

Fig I. The influence of other parameters (in addition to those shown in Fig 3) on the spread of GR protocells (part 2). The representations are the same as those in Fig 3. The values adopted at the three critical change points (red arrowheads) are listed in Table A. See Box A for a comment on the influence.

Table A. The values adopted in the parameter analysis (Figs H and I)

Up-v3	Up-v2	Up-v1	v0	Down-v1	Down-v2	Down-v3
0.2	0.05	0.01	P_{GF}=0.002	0.001	5×10^{-4}	2×10^{-4}
0.05	0.02	0.01	P_{NF}=0.005	0.002	0.001	5×10^{-4}
0.02	0.01	0.005	P_{NPF}=0.002	0.001	5×10^{-4}	2×10^{-4}
0.2	0.1	0.05	P_{PF}=0.02	0.01	0.005	0.002
0.2	0.1	0.05	P_{ND}=0.02	0.01	0.005	0.002
0.01	0.005	0.002	P_{NDE}=0.001	5×10^{-4}	2×10^{-4}	1×10^{-4}
0.05	0.02	0.01	P_{NPD}=0.005	0.002	0.001	5×10^{-4}
0.9	0.5	0.2	P_{GD}=0.1	0.05	0.02	0.01
0.9	0.5	0.2	P_{PD}=0.1	0.05	0.02	0.01
0.1	0.05	0.02	P_{PDM}=0.01	0.005	0.002	0.001
1×10^{-5}	5×10^{-6}	2×10^{-6}	$P_{RL}=1\times10^{-6}$	5×10^{-7}	2×10^{-7}	1×10^{-7}
1×10^{-4}	5×10^{-5}	2×10^{-5}	$P_{BB}=1\times10^{-5}$	5×10^{-6}	2×10^{-6}	1×10^{-6}
100	50	20	F_{DO}=10	5	2	1
0.99	0.98	0.95	P_{AT}=0.9	0.5	0.2	0.1
0.01	0.005	0.002	P_{FP}=0.001	5×10^{-4}	2×10^{-4}	1×10^{-4}
0.9	0.5	0.2	P_{MF}=0.1	0.05	0.02	0.01
0.001	5×10^{-4}	2×10^{-4}	$P_{PLM}=1\times10^{-4}$	5×10^{-5}	2×10^{-5}	1×10^{-5}
0.2	0.1	0.05	P_{TL}=0.02	0.01	0.005	0.002
0.95	0.9	0.8	P_{SP}=0.5	0.2	0.1	0.05
0.99	0.98	0.95	P_{MV}=0.9	0.5	0.2	0.1
0.99	0.98	0.95	P_{FJM}=0.9	0.5	0.2	0.1
0.99	0.98	0.95	P_{PJM}=0.9	0.5	0.2	0.1
20	10	5	F_{PPW}=3	1	0.5	0.2
10	5	2	F_{DE}=1	0.5	0.2	0.1
5×10^{-4}	2×10^{-4}	1×10^{-4}	$P_{NP}=5\times10^{-5}$	2×10^{-5}	1×10^{-5}	5×10^{-6}
0.5	0.2	0.1	P_{NPP}=0.05	0.02	0.01	0.005
0.95	0.9	0.8	P_{NPPP}=0.5	0.2	0.1	0.05
0.99	0.98	0.95	P_{GPP}=0.9	0.5	0.2	0.1
0.001	5×10^{-4}	2×10^{-4}	$P_{CB}=1\times10^{-4}$	5×10^{-5}	2×10^{-5}	1×10^{-5}
0.01	0.005	0.002	P_{CF}=0.001	5×10^{-4}	2×10^{-4}	1×10^{-4}
0.9	0.5	0.2	P_{CD}=0.1	0.05	0.02	0.01
0.9	0.5	0.2	P_{MC}=0.1	0.05	0.02	0.01

Note: The upper portion of the parameters (above the dashed line) is for Fig H and the lower portion is for Fig I. “v0” means the default value; “Up-v1”, “Up-v2” and “Up-v3” means the values adopted at the three change points (one after another; see red arrowheads in the figures) for the case of parameter-turning-up; “Down-v1”, “Down-v2” and “Down-v3” means the values adopted at the three change points for the case of parameter-turning-down.

Box A. On the influence of parameters (for Figs H and I)

Firstly, we note that the default values of the parameters we adopted are almost the best for the spread of protocells containing GR (blue solid circles) – that is, the change of parameter values, either upwards or downwards, could barely improve the level of GR protocells (orange empty circles for "upwards" and cyan empty circles for "downwards") and seldom improve the level of GR molecules (orange dotted lines for "upwards" and cyan dotted lines for "downwards"). This means our initial parameter-exploration is quite successful (see text for the meaning of "parameter-exploration"). Secondly, we see that the spread of GR protocells is robust to the "moderate" change of most parameter values. In the analysis, when turning up or down a parameter, typically a scale of 2 or 2.5 times was adopted, unless the probability might be larger than 1 (see Table A for details). In most cases, the apparent influence on the spread of GR protocells comes only at the third change point, or even never occurs within the changing scope. Thirdly, we comment briefly below on the cases in which the parameter change brings about obvious effects.

(1) For the cases shown in Fig H.

A high probability of non-enzymatic production of glycerophosphates (P_{GF}) is unfavorable because GR's advantage would be weakened. A low probability of nucleotide formation (P_{NF}), which brings about the shortage of the building blocks of RNA, is disadvantageous; likewise, a low probability of nucleotide precursor formation (P_{NPF}) is disadvantageous. On the other side of the coin, a higher probability concerning decay of nucleotides (P_{ND}) and that of nucleotide precursors (P_{NPD}) would also result in the scarceness of RNA's building blocks. A too high probability of RNA's end-decaying (P_{NDE}) may shorten the life span of GR to an extent that the ribozyme cannot sustain in the system through replication. A small factor of degradation outsides protocells (F_{DO}) means the synthesis of RNA within protocells would be short of raw materials. A low probability for an RNA template to attract substrates (P_{AT}) means the template-directed replication of GR would become difficult. A high error rate in the replication of RNA (P_{FP}) is disadvantageous because the heredity of GR is weakened.

(2) For the cases shown in Fig I.

A low probability for substrates aligned on an RNA template to ligate (P_{TL}) is unfavorable because it would also slow down the template-directed replication. A low probability for the separation of a base pair (P_{SP}) is unfavorable because of the difficulty of strand separation in the RNA replication (note that the spread of GR is depressed at the first down-turning point, which means it is quite sensitive to the decline of this parameter). A high probability of protocell-breaking (P_{CB}) is unfavorable because the existence of protocells becomes problematic. A high probability of protocell-fusing (P_{CF}) is disadvantageous because the protocells without GR tend to fuse with those containing GR and thus the GR is "parasitized". Related to this point, a high moving rate of protocells (P_{MC}) is unfavorable because this would tend to bring the protocells without GR adjacent to GR protocells and enhance the likelihood of the cell-fusion.
