# Supplementary material for: From experimental clues to theoretical modeling: Evolution associated with the membrane-takeover at an early stage of life
Source: PLoS Comput Biol. 2025 Jun 13;21(6):e1012763. doi: 10.1371/journal.pcbi.1012763 (PMC12201655; doi:10.1371/journal.pcbi.1012763)
Supplement: Attachment — Submitted filename: Response to Reviewers.docx [file pcbi.1012763.s003.docx]

We thank the reviewers for their comments on our paper. The following is our response to their opinions and suggestions.

**Reviewer #1:** *This paper has strong merits and some weakness. The strength is that it attempts to get as close to experimental data as possible, without losing site of the evolutionary dimension. For the majority of the parameter values the Authors perform a careful choice. Nice work!* --- Thanks.

*TECHNICAL ISSUES:*
1. *There are reactions that can be catalyzed by specific enzymes. The uncatalyzed reactions are reversible. If so, the catalyzed reaction should also be reversible and faster in BOTH directions, hence the equilibrium constant for the same reaction should be the same. I think this will not lead to a qualitative change but will certainly make a quantitative difference. Since membrane growth and replication are sinks for the building blocks, the system will work, but less efficiently. The "protocells" are open systems, so the equilibria can shift with the degree of flow through the system, but openness does not turn reversible reactions into irreversible ones. This is a consistency problem. I suggest the Authors do a control for ONE of the reactions to see the magnitude of the quantitative change. If there are surprises they have to redo everything, I am afraid.*
--- There is a misunderstanding here. In the model, just as it is stated in Methods, “the energy problem is here not considered explicitly”, the precursors are actually activated ones in the synthetic reactions (as a reactant) but inactivated ones in the decay reactions (as a product). For example, the synthesis and the decay of nucleotides ar here not reciprocal reactions (as shown in Fig. 7). In other words, the uncatalyzed synthetic reactions are actually irreversible – hence, the catalyzed synthetic reactions are also irreversible (or else, one may imagine that the synthetic reaction was actually coupled with an irreversible reaction involving a high-energy factor like ATP, but the high-energy factor is omitted in the present model). To avoid this misunderstanding, we have added a relevant explanation following that paragraph concerning the energy problem: “Another issue, which is associated with the energy problem, concerns the synthetic reactions and the corresponding decay reactions of building blocks. In the model, the precursors (Npp, Np and Gp as depicted in Fig. 7) are actually activated ones in the synthetic reactions (as a reactant) but inactivated ones in the decay reactions (as a product). In other words, a decay reaction here is actually not the reverse reaction of the corresponding synthetic reaction – both of them are actually irreversible. Notably, as a consequence, the ribozyme-catalyzed synthetic reactions (related to *P_NPFR_*, *P_NFR_* and *P_GFR_*) in the model are irreversible.” Since we just intend to model the ribozyme-catalyzed reactions as irreversible reactions, “do a control … to see the magnitude of the quantitative change” should be unnecessary. As to the “sinks for the building blocks” mentioned by the reviewer, we agree completely. For example, nucleotides formed would be used in the template-directed synthesis of RNA, and glycerophosphate formed would be used in the synthesis of PL --- the removal of these products would actually enhance the irreversible reactions. But this content is not directly related to our topic here, and we would like not to discuss it in the paper.

*2. A big simplification is how they treat RNA strand separation. It is still an open problem, unless the molecules are very short (as for von Kiedrowski origonucleotide replication). For most riboyzmes strand separation just will NOT work in the way they are modelling the process. 3D spontaneous folding might help somewhat, but it remains true that the totally annealed, complementary strands forming a duplex IS the STABLEST form (energy minimum). Having said this I think they can leave this as it is, since the focus of the paper is something else. But a remark on this is in order in the Discussion.*

--- Yes, thanks. We have added the following remark concerning this issue: “It should be noted that in reality, the chain of a ribozyme may have been substantially longer than what we assumed in the model, and thus it is likely that the separation of the two strands was quite difficult. Although spontaneous self-folding of single chains might be of some help, RNA strand separation still seems to be an open problem in the field. Since the focus of the paper is not on this, here we do not intend to analyze it in detail.” But please note that to be more focused on our topic in the Discussion section, this remark has been added in the relevant part of the Methods section where the RNA strand separation is described.

3. *They define short necessary motifs for the three enzymatic functions. The small size is for computational reasons, but then it is very easy to go from one enzymatic function to another. Longer and more dissimilar motifs would require long and big simulations, for sure. But this brings me to another, interesting issue: the role of protocell fusion. The latter could turn out to be critical with long motifs, since then fusion could be necessary for bringing pieces of the genome together (a bit like the Fisher-Muller theory of sexual recombination). My concrete suggestion is to repeat at least some of the runs by setting the fusion rate to zero to see what happens. Discuss the results.*

-- Yes, the reviewer’s opinion is reasonable. Cell fusion may be a factor strengthening the spread of protocells with multiple genes. According to his suggestion, we turned off the fusion rate midway for the system of NR-GR-NPR protocells and NR-GR-TR protocells --- that is, based upon the cases shown in Fig. S6, at step 1.4×10^6^, *P_CF_* is set to zero instead of turning off the function of NPR (in *a*) and TR (in *b*). We indeed found that the NR-GR-TR protocells drop off, but NR-GR-NPR protocells are not influenced (Fig. R1, note the solid circles and the solid lines).

**Fig. R1** The influence of turning off the cell fusion rate. The cases are the same as those in Fig. S6 except that at the critical point (step 1.4×10^6^), it is PCF that is set to 0 instead of *P_NPFR_* (in **a**) and FTR (in **b**).

Then, to see the robustness of this result, we changed the critical point to step 1.3×10^6^ and 1.5×10^6^ (Fig. R2 and R3, respectively), and found that in both cases, the spread of the multiple gene-protocells is stable. There seems to be more detailed mechanisms involved in this issue (perhaps cell fusion is not necessary but can render the spread of the multigene protocells more stable to some extent). Considering that this issue is not directly associated with our topic, we do not want to make further discussion in the paper (but with the source code available publicly, one may trace into any related issues of interest to him).

**Fig. R2** The situation is the same as that shown in Fig. R1 except that the critical point is step 1.3×10^6^.

**Fig. R3** The situation is the same as that shown in Fig. R1 except that the critical point is step 1.5×10^6^.

*CONCEPTUAL:*

*The title says "from experimental clues to theoretical modelling". Well said, but in historical reality it ought to something like "from theory to experiment to theory". They are missing two crucial theoretical references.*

*The first is* *Szathmary (2007). Coevolution of metabolic networks and membranes: the scenario of progressive sequestration. This paper predates that of Szostak (Ref. 9. in the paper). That paper discusses the appearance of ribozymes, progressive decrease of permeability, the selection for "enzymatization" of previously uncatalyzed reactions, and selection for permease function (referring to an experimental RNA case). At any rate this paper is too close to be neglected. In reality one could say that the present manuscript has one theoretical root and one experimental root, but the former has been neglected. This should be remedied in the Introduction already.*

--- Yes, thanks for mentioning this paper. The paper, which envisions a progressive enzymatization process and the accompanying coevolution of the membrane (with its permeability gradually decreasing), is impressive. We agree that it is closely related to the current paper. However, it seems inappropriate to be regarded as the theoretical base of Budin and Szostak’s experimental work. First, the experimental work proposed the plausibility of the membrane-takeover owing to the role of phospholipid content in stabilizing the membrane, which is not addressed in Szathmary’s paper. Then, the experimental work suggested that “membrane evolution may have driven the metabolism evolution and permease evolution”, while the Szathmary’s paper holds a viewpoint of “metabolism evolution may have driven the membrane evolution and then the permease-evolution”. We choose to mention Szathmary’s paper in our discussion: “It should be noted here that there was an early theoretical paper [47] dealing with a topic related to the idea proposed in Budin and Szostak’s experimental paper. In that paper, Szathmary suggested a scenario in which the membrane would have evolved towards becoming more impermeable to retain in protocells the building blocks synthesized by enzymatic reactions, and as a consequence, functions that favor across-membrane transportation may have then emerged. That is an evolutionary cascade from metabolism to membrane to transport mechanisms, while the experimental paper proposed an evolution order from membrane to metabolism and transport mechanisms. Certainly, the former scenario is another interesting one deserving modeling study in future”.

*The second paper that includes also quantitative modelling is Piedrafita et al. (2017) Permeability-driven selection in a semi-empirical protocell model: the roots of prebiotic systems evolution. Sci. Rep. 7, 3141. This is also a very serious neglect. That paper offers useful considerations as well as a dynamical model (although RNA replicators are not modelled). Compare it carefully with your approach in the Discussion, and acknowledge key items that predate the present manuscript. Pay attention to the division problem that is just "written into" your present code.*

--- Yes, this paper is also related to the current topic. We discussed it just following the discussion of the first paper (Szathmary, 2007): “Another related paper concerns a more recent modeling study [48], in which the evolution driven by the coupling of membrane and “proto-metabolism” was investigated. However, by “proto-metabolism” they mean that no enzymatic reaction is involved. That is, the genetically-encoded mechanism is not included in the scenario, and the relevant evolution is a “pre-Darwinian evolution". Compared with our model, the model used in this study is much more abstract, but notably, its method for dealing with the growth and division of protocells is more concrete than ours (i.e., considering the actual balance between vesicular surface area and internal volume). Additionally, their model is a “semi-empirical” one – i.e., with some assumptions just based on their own experimental data. This represents an effort to combine experimental work and theoretical work directly (see below for a discussion of the combination of experimental and theoretical studies in this field with a broader sense).”

**Reviewer #2:** *In this manuscript, Monte Carlo simulations are set to simulate competing growth of protocells based on phospholipid metabolism, which was proposed based off of experimental studies by Budin and Szostak. In this model, catalyzed phospholipid synthesis would have driven the growth of protocell populations at the expense of others that produced fewer and no phospholipids. The mechanisms identified in that study were that phospholipids slowed the desorption rate of fatty acids from a membrane and that they themselves are insoluble, so they also proportionally increase adsorption vs. desorption surface area. This manuscript identifies both of these effects as potentially contributing to competitive protocell growth. They then explore other aspects of the emergence of phospholipid metabolism – increased selectivity for other relevant enzymes to maintain phospholipid catalysis and transporters to counter the reduced permeability rate of phospholipid membranes.*

--- Yes, the reviewer’s description of our study is quite right, thanks.

*Overall, this is an interesting study, because this model for phospholipid-based competition has never been systematically investigated after its proposal. I would say the results here not surprising, but several of them are relevant, for example in identifying that both models of phospholipid effects on fatty acid dynamics could be relevant for competition. I do think the writing and presentation of the paper and its data could be substantially improved -making its data and figures more accessible to a broader audience while implementing a higher technical level of writing in the results and discussion.*

--- Yes, our aim is just to systematically investigate the interesting proposal by computer modelling, thanks. See below for our response to the points raised by the reviewer.

*1) The authors treat acylation reactions as trivial, with the limiting step to forming a PA being synthesis of glycerolphosphate. This is not the case; acylation is endergonic and requires a fatty acid to be activated first. Recent work they cited showing abiotic phospholipid synthesis all requires pre-activated fatty acid thioesters. Also, acylation of a naked glycerol to form a DAG also has the same growth phenotypes as PA, since the insolubility of the DAG also restricts efflux. Thus, I don’t understand the focus on a glycerolphosphate synthase ribozyme as the key step in this process.*

--- Yes, acylation of fatty acid is endergonic and we don’t mean that this reaction can occur readily free of an energy-driven mechanism. Actually, as we have explained in Methods, “the energy problem is here not considered explicitly”, the activation of fatty acids is assumed implicitly. As previously mentioned in our response to Reviewer #1 regarding the irreversibility of the synthetic reactions within our model, one may imagine that a high-energy factor like ATP may have been involved (in the formation of PA) but is omitted here for simplification. If the energy issue is of no problem, the acylation reaction is indeed more likely to occur in a non-enzymatic way, and the formation of glycerophosphate may have been the limiting step – as elaborated in the paper. In fact, as mentioned in Discussion, another reason why we did not adopt a ribozyme catalyzing the acylation reaction is that RNA is likely difficult to cope with reactions occurring on the membrane because of its polar skeleton – in reality, this function may have emerged after the advent of proteins. To strengthen this point, we added: “For example, in modern cells, the acylation reaction to form phospholipids (on the membrane) requires preexisting membrane-embedded enzymes [43,44]”. Likewise, acylation of a naked glycerol to form a DAG should also involve membrane-bound enzymes. In other words, if we adopt DAG as the target emerging in the membrane-takeover, this evolutionary event should not have occurred in the RNA world. After all, in our modeling, the glycerophosphate synthase ribozyme (GR) here is merely the representation of a ribozyme favoring the synthesis of phospholipid – in fact, in another model version (not presented in this paper), we assume that there is a ribozyme that catalyzes the acylation of fatty acids instead – this ribozyme favoring the formation of PA may also spread in the model system.

*2) Parameter for various functions (like fatty acid desorption rates) could be better constrained by experimental data, which exists. I understand the appeal of sweeping large ranges of parameter space to look for trends, but in this case we know some of these rates.
3) A good example of the above is the PFLM vs PPLM values assigned. A fatty acid is set to desorb 20 times slower than a phospholipid – in realty this more is more like a million fold or more based off of published data.*

--- Yes, due to our limited knowledge about prebiotic environments and chemistry, it is usually difficult to justify the concrete value-setting of parameters in this area. Thus, the approach of “sweeping large ranges of parameter space to look for trends”, which we termed “reverse modeling” is often necessary (see Ref. 15). Certainly, we agree that experimental data can assist in the setting of parameters for modeling, as already mentioned in the “The setting of parameters” subsection of Methods. Regarding the example mentioned by the reviewer, first, we want to emphasize that the factor *F_PL_* (the factor of phospholipids' influence on amphiphiles leaving the membrane) should be taken into account. That is, RPM (the ratio of phospholipids in the membrane) also affects the desorption rate of phospholipids themselves. As a result, the actual probability of a phospholipid molecule detaching from a pure-phospholipid membrane is *P_PLM_*/(1+ *F_PL_*) (assuming the osmotic pressure effect is not considered, see the subsection of Methods “Some detailed mechanisms concerning the implementation of the model”). If *F_PL_* adopts a large value, the relevant difference of desorption rates between fatty acids (from the fatty acid membrane) and phospholipids (from the phospholipid membrane) could be significantly greater than the difference between *P_FLM_* and *P_PLM_*. Additionally, the difference of desorption rates between the two lipids may be influenced by environmental factors such as temperature, pH and ionic strength.

**Fig. R4.** The effect of setting a much smaller *P_PLM_*. The representations are the same as those in Fig. 3. The cases are actually an extended version of Fig. S8-2-*P_PLM_*, in which a fourth critical change point is introduced at step 3.5 ×10^4^ (the most right red arrow). At this point, *P_PLM_* is set to 1×10^-8^ for the case of parameter-turning-down (the cyan symbols).

Anyway, in this paper, we have conducted the parameter analysis concerning their influence. It was found that, apart from those “key parameters” (Fig. 3), the result regarding the spread of “GR”, in general, is fairly robust against “moderate adjustments” of the other parameters (Fig. S8-1 and S8-2). To address the concern raised by the reviewer, we extended the case concerning *P_PLM_* (Fig. S8-2-*P_PLM_*) by turning down the parameter to a level of 1×10^-8^ at a fourth critical change point (step 3.5×10^6^), and saw no obvious influence (Fig. R4, the cyan symbols). Considering the default values of *F_PL_*=5 and *P_FLM_*=0.002, the relevant difference of desorption rates between fatty acids and phospholipids here is actually 1.2×10^6^-fold. In addition, notably, if we have assumed a relatively small difference between the two kinds of lipids, that is just a conservative assumption for this modelling – logically, a greater difference may have even promoted the emergence of the function that favors phospholipid synthesis.

Because these contents are somewhat difficult to incorporate into the main text of our paper, we would like to leave them here and take advantage of the journal’s policy of making the review documents publicly accessible. Thanks.

*4) I find the figures 2,3,5,6 to be extremely hard to parse for a broad audience*

--- Yes, thanks. We have made some modifications in the legends of these figures.

For Fig. 2, a sentence explaining both the meaning of the upper panel and lower panel is inserted following the title of the figure: “For each subfigure, the upper panel shows the trend of protocells containing specific RNA species while the lower panel demonstrates the trend of the total molecule number of the relevant RNA species in the system.” (Certainly, the words that only explain the lower panels in the origin version are deleted).

For Fig. 3, a similar explanation is added: “For each subfigure, the upper panel shows the trend of GR protocells while the lower panel demonstrates the trend of the total number of GR molecules in the system.” Additionally, to be clearer, the legend interpretations are changed to: “Legends: Cgr — GR protocells with all parameters adopting default values; Cgr-para-up — GR protocells with the relevant parameter increasing; Cgr-para-down — GR protocells with the relevant parameter decreasing; gr — GR with all parameters adopting default values; gr-para-up — GR with the relevant parameter increasing; gr-para-down — GR with the relevant parameter decreasing (the legends apply to all the subfigures).”

For Fig. 5, an explanation is also added: “For each of the two subfigures, the upper panel shows the trend of relevant protocells while the lower panel demonstrates the trend of RPM (the ratio of phospholipids in the membrane) for these protocells (averaged for each type of protocell).” Correspondingly, the interpretation of RPM is simplified as: “RPM for a protocell is calculated as 2×*p_num_*/(2×*p_num_*+*f_num_*), where *p_num_* and *f_num_* denote the number of phospholipids and that of fatty acids on the membrane respectively (note that a phospholipid molecule has two non-polar tails whereas a fatty acid has one).”

For Fig. 6, the corresponding explanation is: “For each of the two subfigures, the upper panel shows the trend of relevant protocells while the lower panel demonstrates the trend of relevant RNA species.” Additionally, for a clearer appearance, the legend interpretations are changed to: “The legends identical to those included in Fig. 2 have the same meaning, and additional ones are as follows: Cnrgrnpr — protocells containing NR, GR and NPR; Cnrgrtr — protocells containing NR, GR and TR; npr — NPR; tr — TR.”

*5) In the introduction, there is an incorrect statement/simplification of fatty acids and phospholipid systems (lines 47-52). Fatty acids are more dynamic because they are soluble and contain small, less polar head groups. So exchange of fatty acids and flip flop across leaflets is much faster, and this can be utilized for growing membranes. The citations provided showing prebiotically plausible mechanisms of phospholipid synthesis and new interesting phenomenon in phospholipid GUVs does not change that.*

--- Yes, we admit that the expression here is inappropriately simplified and have rephrased the relevant paragraph as: ‘Traditionally, as a further argument for the scene of “fatty acids first”, it is emphasized that these single-chain lipids are more dynamic – **with small, less polar head groups, their exchange between leaflets (the so-called flip-flop) is much faster**, thus favoring the spontaneous growth and division of protocells [1-3]. But evidence has now accumulated to show that phospholipid-based membranes are also sufficiently dynamic for growth and division [4,6-8]. **Therefore, this argument against phospholipids being the first membrane lipids seems no longer that strong.** However, notably, if the scene of “fatty acids first” is true, these findings on phospholipid-based membranes actually imply that the membrane-takeover from fatty-acid membranes to phospholipid membranes could have occurred quite early – well before the advent of complex forms like modern cells, which have specific functions regarding cellular growth and division.’ Please pay attention to the bold words.

*6) The references study, which this manuscript is based on, has only two authors, so the correct citation in the is not Szostak and coworkers (plural). Budin and Szostak as the two authors or the Szostak group generally is more appropriate.*

--- Yes, we have corrected the expression accordingly, thanks.

**Reviewer #3:** *This paper considers various possible ribozymes that have a positive influence on the growth of protocells. The model shows that cells containing any of these ribozymes can be selected by evolution. The model is well motivated and the results are quite clear. I think the work is definitely publishable, although I have several suggestions for improvements and clarification.* --- Thanks.

*The paper investigates the possible transition from membranes composed of fatty acids, to membranes composed of phospholipids. It is assumed that phospholipid synthesis is catalyzed by ribozymes. This is plausible, but maybe there could be other scenarios. In other words, could it be possible to have phospholipid membranes prior to the existence of RNA replication? Do we really know that growth and division of protocells must have been controlled by ribozymes? If we preferred a metabolism-first view of life, could we not also get a takeover of fatty acid membranes by phospholipids?*

--- Yes, there could have been non-enzymatic synthesis of phospholipids, as also considered in our model (related to *P_GF_* and *P_PF_*). However, because of the inefficient nature of the uncatalyzed synthesis, the quantity of phospholipids would be rather limited, and RPM (the ratio of phospholipids in the membrane) of the final protocells would be very low (one may refer to the stage before the rising of GR, either in Fig. 5a or 5b). Note that even with the emergence of GR (*P_GFR_* is much higher than *P_GF_*), RPM of the relevant protocells could be not very high (Fig. 5), with the takeover at a limited level. As analyzed in our study, this may be attributed to the subsequent inefficient non-enzymatic reaction of glycerophosphates with fatty acids on the membrane (*P_PF_*). Indeed, when we assume a high rate for the non-enzymatic formation of phospholipids (i.e. *P_PF_*) in the midway, the ratio of phospholipids rises to a rather high level immediately (Fig. S2). This result implies that in reality, it should be the later emergence of a ribozyme or enzyme favoring this bottle-neck reaction that may have ultimately taken the membrane-takeover towards a more thorough degree. On the other side of the coin, logically, we cannot expect a prominent takeover of fatty acid membranes by phospholipid membranes before the emergence of Darwinian evolution unless the non-enzymatic phospholipid synthesis (preferably each step in the “pathway”) could have been rather efficient.

In order to mention the influence of non-enzymatic phospholipid synthesis before the advent of ribozyme favoring phospholipid synthesis, we have added in Discussion after the stepwise membrane-takeover is explained: “That is, the membrane-takeover may have been a stepwise process in evolution. Notably, even before the emergence of enzymatic function favoring the synthesis of phospholipids, there would still have been a level of phospholipid content in the membrane, though marginal (one may refer to the stage before the rising of GR, either in Fig. 5a or 5b). This can be attributed to the non-enzymatic synthesis of phospholipids (quite inefficient, related to *P_GF_* and *P_PF_* in the model).”

*The rules used for formation and growth of membranes need to be explained more carefully. On Line 449, there is a minimum number of lipid molecules L_AM required on one lattice site to make a new cell. I assume that a cell instantaneously assembles as a single event. What if there are more than L_AM molecules on the lattice site? Are the additional molecules included in the membrane or are they assumed to be inside the cell? What about any molecules of other kinds that are on the lattice site at the time the cell forms? Are these immediately assumed to be inside the cell?*

--- Yes, we have modified the corresponding expression as: “In a grid room, if the quantity of amphiphiles (determined by the number of tails: a fatty acid is regarded as one, while a phospholipid is regarded as two) **is larger than** *L****_AM_***, they may aggregate at the boundary of the room and form a membrane (*P_MF_*), thus creating a protocell. **During the assembly process, all of the amphiphiles are incorporated into the membrane, while other molecules are located inside the protocell**.”

*It seems that if two cells fuse, this creates a single cell on one lattice site only. In other words,* *a single site can hold one cell of arbitrarily large size. Is this correct?*

--- Yes, that is right (refer to Fig. 7). This assumption is for simplification of the modeling. But please note some compensatory considerations for such a simplification – for example, in the events regarding the leaving of amphiphiles from a membrane as well as those concerning the permeation of materials into a protocell, where the membrane surface area and the protocell volume are evaluated according to the amount of amphiphiles in the membrane (see the subsection “Some detailed mechanisms concerning the implementation of the model” in Methods).

*When fatty acids join or leave an existing membrane, do these come from neighboring lattice sites, or from the same site as the cell? If they come from the same site, are they assumed to be inside or outside the cell before they join the membrane? The squares representing the membranes are drawn around the whole lattice site. This suggests that all molecules on one site must be inside the cell, in which case new lipids must be added from neighboring cells. Line 433 says “see below for the situation of encountering a membrane” – but I cannot find where this is described.*

--- When amphiphiles join an existing membrane they may either come from neighboring lattice sites or from the same site – within the cell (that is, all molecules on one site with a surrounding membrane are inside the cell). In fact, these joining events can be included in the situation concerning a molecule’s movement when it encounters a membrane. Therefore, we have modified the sentence mentioned by the reviewer as: “A molecule may move to an adjacent room (the related probability: *P_MV_*) if there is no membrane in that direction, **and may join or permeate through the membrane if there is one (amphiphiles and glycerophosphates may join while other molecules may permeate through, see below for details regarding relevant probabilities**).” Likewise, when the lipids leave a membrane, they may either enter a neighboring lattice site (randomly in four directions) or enter the same site – into the cell itself.

*For a fatty acid solution with concentration C, there should be some critical aggregation concentration C* above which membranes form spontaneously. For C < C*, there should be almost entirely dissolved molecules and no vesicles. For C > C* there will be a certain number of vesicles and a remaining number of dissolved molecules with a concentration equal to C*. Does the current model have this property? It would be useful to run a simulation with only fatty acids and no other molecules, and to measure the concentration of dissolved molecules and vesicles as a function of the total number of fatty acids, in order to show that a critical concentration exists.*

--- Yes, the current model has this property. Actually, the *C** is affected by the relative value of two probabilities: *P_FLM_* (fatty acid leaving the membrane) vs *P_FJM_* (fatty acid joining the membrane). Fig. R5a shows that accompanying the increase of *P_FLM_* (with *P_FJM_* in its default value 0.9) the protocells in the system decrease and the dissolved fatty acids in the system increase. When *P_FLM_* adopts a value of 0.2 (after the third red arrow), the protocells decrease to 0 and the free fatty acids rise to the total number of fatty acids in the system (*T_FB_*=50000), which means that the total number of fatty acids does not reach the *C** to support the existence of any protocells in the system. Similarly, accompanying the decrease of *P_FJM_*, (with *P_FLM_* in its default value 0.002) the protocells in the system decrease and the dissolved fatty acids in the system increase. When *P_FLM_* adopts a value of 0.005 (after the third red arrow), the protocells decrease to 0 and the free fatty acids rise to the total number of fatty acids in the system (*T_FB_*=50000), which means that the total number of fatty acids does not reach the *C** to support the existence of any protocells in the system.

**Fig. R5** The influence of two key probabilities on the critical aggregation concentration (*C**) of fatty acids. In the beginning of the simulation, only fatty acids are introduced into the system (i.e., without other molecules). With the inoculation of an “empty” fatty-acid protocell at step 1×10^3^, the protocells (the upper panels) spread and the free fatty acids (the lower panels) in the system decrease. For (***P_FLM_***), the default value 0.002 is turned up to 0.01, 0.05 and 0.2 at the three points of change (red arrows), respectively. For (***P_FJM_***), the default value 0.9 is turned down to 0.1, 0.02 and 0.005 at the three points of change, respectively. All the other parameters adopt their default values. The number of fatty acids is used as a relative ruler of their concentration since the system’s area is fixed.

Note that for our two-dimension system, the concentration of fatty acids means the number of fatty acids divided by the area of the system. But no absolute value has been assumed for the latter here, hence, the number of fatty acids is used as a relative measure of their concentration. For example, in Fig. R5-*P_FJM_*, for the situation demonstrated between the second arrow and the third arrow (in which *P_FLM_*=0.002 and *P_FJM_*=0.02), the *C** is about 20000/*A_S_*, where *A_S_* denotes the area of the system.

*If I understand correctly, membrane growth in this model requires diffusion of a dissolved lipid from one site onto a neighbouring site that already contains a cell. The rate of addition of molecules to the membrane therefore appears to be independent of the current membrane area. I am assuming that each lipid in a membrane has the same probability of leaving the membrane, so the rate of molecules leaving the membrane is proportional to the current area of the membrane. If there were no cell division or fusion in the model, cells would reach a steady state size where loss of lipids balances addition. In reality, cells have different membrane areas. We would expect the rate of addition of molecules to a cell of area A to be proportional to A. If both addition and loss of lipids were proportional to current area, then the area would either increase or decrease exponentially, according to whether addition or removal were larger. This seems to be an important difference.*

--- Yes, in the model, membrane growth requires diffusion of a dissolved lipid from one site onto a neighboring site that already contains a cell, and the rate of addition of molecules to the membrane is indeed independent of the current membrane area. Therefore, the simplification of assuming “a single site can hold one cell of arbitrarily large size” (as already mentioned above), seems an intrinsic shortcoming in this aspect, since any protocell, despite its size, only has a four-direction surrounding in the model. In other words, it doesn't take into account the fact that “the larger surface area of a membrane, the greater chance for the membrane to encounter lipids in the surroundings, which might join it”. A compensation to this issue may be to assume that a lipid in the surroundings, when encountering a protocell, may have a greater chance to join its membrane if the membrane has a larger surface area. But this compensation seems to be a bit too “*ad hoc*” so that it might be difficult for one to judge about its potential influence. In fact, fortunately, the simplification is a conservative assumption for our topic here – that is, if a growing membrane has a chance to grow faster, the ribozyme favoring phospholipid synthesis would enjoy a greater selective advantage to spread by favoring the growth of protocells that contain it.

Accordingly, we have added a comment to this point in the “Some detailed mechanisms concerning the implementation of the model” subsection of Methods: “It should be noted that, for the sake of simplicity in the model, we assume that a protocell occupies a grid room. This may represent a shortcoming regarding the joining of amphiphiles to the membrane, since any protocell, despite its size, is only surrounded on four sides (of a grid room). In other words, it does not take into account the fact that “the larger the surface area of a membrane is, the greater the chance is for the membrane to encounter amphiphiles in the surroundings that might join it”. Fortunately, this simplification is a conservative assumption for our topic here – that is, if a growing membrane has a chance to grow faster, the ribozyme favoring phospholipid synthesis would enjoy a greater selective advantage to spread by favoring the growth of protocells that contain it.” Thanks for mentioning this issue.

Regarding the difference mentioned by the reviewer, in the model system, there is indeed a negative feedback for the growth of the protocell, and in reality there should not be. But that does not mean that in reality there would be a positive feedback for the growth of the protocell. In fact, since a larger membrane surface means just a larger exchange surface – i.e., for both the joining and the leaving of lipids (as stated by the reviewer, “*both addition and loss of lipids were proportional to current area*”). In other words, it seems unlikely that the growth of membrane area would adopt an exponential mode since a larger surface means both that more lipids have a chance to join the membrane and that more lipids have a chance to leave the membrane.

*The factor y on line 533 represents the osmotic pressure effect observed in ref 60, where it is observed that cells containing RNAs with no function gain lipids at the expense of empty vesicles. This experimental effect appears important, and it would be useful to demonstrate it in the model – i.e. to show that if you start with a mixture of cells containing random non-functional RNA templates and cells containing no RNA, the cells containing RNAs will multiply and the empty cells will die.*

--- Yes, here we demonstrate the effect in our modeling system (Fig. R6). In this case, we set *T_GPB_*=0 to avoid the interference of phospholipids. When an empty protocell (containing no RNA) is inoculated at step 1×10^3^, the protocells spread. Then, at step 1×10^4^, ten of the empty protocells are each inoculated with one control molecule (non-functional). We set *P_RL_*=0 to ensure no RNA molecule can be generated through random ligation of nucleotides, and *P_FP_*=0 to ensure the control species would not mutate in its replication. Therefore, the protocells containing the control species can serve as a representation of all protocells containing RNA, while the protocells without the control species can represent all protocells that do not contain RNA. We set *P_CF_*=0 to ensure that no event of cell fusion can occur – thus the two kinds of protocells have a relationship of purely competitive. The result shows that, indeed, “the cells containing RNAs will multiply and the empty cells will die”. Since this issue is not the focus of the present study and this is not the first time we have introduced the osmotic pressure effect in our modeling work (see Refs. 22-24), we would like to leave this content here and take advantage of the journal’s policy of making the review documents publicly accessible. Thanks.

**Fig. R6** The influence of the osmotic pressure effect on the competition between protocells. Legends: C_0 — protocells not containing the control RNA species; Cctl — protocells containing the control RNA species; ctl — the control RNA species. With the inoculation of an empty protocell (protocell containing no RNA) at step 1×10^3^, the protocells spread. Then, at step 1×10^4^, ten empty protocells are selected (arbitrarily), each inoculated with one control molecule. *T_GPB_*=0, *P_RL_*=0, *P_FP_*=0, and *P_CF_*=0. All the other parameters adopt their default values.

*The functional forms for the factors y and z on line 534 seem very qualitative. I agree that you want the leaving rate of the fatty acids to decrease when the fraction of phospholipids goes up and you want the leaving rate of the lipids to decrease when there is an osmotic pressure (or the addition rate to increase). But there is no “physics” in these functions. I would have preferred it if these functions could be derived from considerations of the energetic stability of the membrane, rather than just making up arbitrary functions. I also note that the scaling b^3/2 on line 540 is only true if the cell is spherical. In reality cells cannot be spherical. Spherical cells cannot divide because there would be insufficient lipid to enclose two smaller spheres of half the volume. There is no physics of cell division in this model – it is supposed that there is a constant probability of division of all cells, but in reality there must be some dependence on volume, area and shape of the cells. I see that the main point of this paper is to demonstrate selection for ribozymes with useful function. I expect that this will occur whatever the details of the cell division process, but I am pointing out that a lot of interesting aspects of cell division behaviour are ignored in this model.*

--- Firstly, there is a misunderstanding here – the assertion “*it is supposed that there is a constant probability of division of all cells*” is not the true. Please note our explanation on the division of protocells in the “Some detailed mechanisms concerning the implementation of the model” subsection of Methods: “The probability of protocell division is assumed to be *P_CD_* × (1 – 2 × *L_AM_*/*b*), where *b* is the quantity of amphiphiles within the membrane. When *b* is no more than twice that of *L_AM_*, the probability is no larger than 0, i.e., the protocell cannot divide. This assumption considers the fact that the larger the protocell, the more probable it would divide, on account of the physical instability.”

Secondly, we admit that for most points mentioned here by the reviewer, we merely adopt a quite qualitative mode. This is for the sake of simplicity in the modeling, and in fact, we have found our modeling is quite robust to these qualitative assumptions. For example, the osmotic pressure effect could be well presented by the factor y in the simulation, as demonstrated in Fig. R6. As another example, in this study, the probability of the movement of an RNA molecule is assumed to be *P_MV_*/*m*^1/2^, where *m* is the mass of the RNA, relative to a nucleotide. This assumption represents the consideration of the effect of the molecular size on the molecular movement. The square root was adopted here according to the Zimm model, concerning the diffusion coefficient of the polymer molecules in the solution [68]. However, in our early studies (Refs, 22 and 23), the Zimm model is not referred and a cube root was used instead – actually, it is not until we did the work associated with Ref. 24 when we noticed the Zimm model. Practically, in the results of simulation, we found no obvious difference for these different assumptions. As for the cell division, we also agree that most of the detailed physics is omitted, mainly owing to the assumption here regarding one protocell occupying one grid room, as mentioned already. The shape of the protocells cannot be considered and the volume and area can only be considered implicitly. Note that the cell division problem is also commented by Reviewer 1#, who mentioned the merit of another study in this aspect (*Piedrafita et al. 2017*). Anyway, we have indeed adopted simplifications in quite a few aspects, especially considering that the present model system is fairly complex. We believe or expect that these simplifications would not interfere with the key mechanisms related to the current topic.

*The model has rules for RNA polymerization and templating. I am presuming that these reactions happen both inside and outside cells. In other words, there is nothing that differentiates the inside and outside of the cell. In reality we would like a cell to have an active autocatalytic reaction inside, while the environment remains inactive. We do not want autocatalysis to start spontaneously in the environment otherwise there would be no difference between inside and outside. See recent paper of Sanders et al https://journals.aps.org/pre/abstract/10.1103/PhysRevE.111.014424 . In that paper we have shown that if templating is fast, but spontaneous synthesis of oligomers from monomers is slow, then it is possible for a cell that begins with oligomers to maintain templating inside the cell while there is no initiation of templating outside the cell. It takes a rare event to create a cell that contains sufficient oligomers to initiate the templating process, but once initiated, RNA synthesis continues. Thus RNA templating can play the role of a metabolism that maintains the difference between the inside and outside of a cell. For the model used in the current paper I am therefore interested in whether templating can initiate if there are only monomers to start with. It would seem reasonable to set polymerization and templating rates such that templating is maintained inside cells that begin with oligomers but is not initiated outside cells where there are no oligomers.*

--- Yes, in the current model, the reactions concerning RNA polymerization and templating happen both inside and outside cells. However, there is something that differs between the inside and outside of the cell. A factor, *F_DO_*, is assumed to affect the decay of nucleotides, the nucleotide residue decay at RNA’s chain end and the chain breaking of RNA outside protocells (See the subsection “Some detailed mechanisms concerning the implementation of the model” in Methods). In other words, due to the higher water activity outside the protocells, we assume that nucleotides and RNA would be easier to degrade there (*F_DO_* >1). In fact, we found the spread of RNA-based protocells is relatively sensitive to this assumption – for example, when *F_DO_* is changed to 1, which means that there is no difference between the inside and outside of protocells in this aspect, the spread of GR protocells collapses (Fig. S8-1-*F_DO_*). Because the situation mentioned in the reviewer-cited paper is about RNA-based protocells without ribozymes, a case more relevant to the reviewer’s concern is the one shown in Fig. R6, which involves protocells containing a control RNA species. Therefore, based on that case, we change *F_DO_* from its default value 10 to 1 after the spread of these RNA-based protocells, and found that the protocells’ spread also collapses and the control RNA species spread outside the protocells (Fig. R7). That is to say, if there is no difference in nucleotides and RNA degradation between the inside and the outside of the protocells, the template-directed RNA replication cannot be maintained within the protocells. According to the logic explained in the reviewer-cited paper, we reasoned that this is because in our model, nucleotides may nucleate on the template to form a primer, and thus a primer is not a “catalyst” – consequently, the template-directed RNA replication does not adopt a second-order autocatalytic mechanism. However, we are not sure whether this conclusion is correct because their model and our model seem quite different.

**Fig. R7** The influence of *F_DO_* on the template-directed RNA synthesis in protocells. The case is the same as that shown in Fig. R6, except that at step 1.5×10^5^, *F_DO_* is changed from its default value 10 to 1. After the change point (the red arrow), the protocells containing the control RNA species decline and empty protocells rise, which means the control RNA species replicates outside the protocells instead of inside the protocells (even reaching a higher level, perhaps because without the membrane, raw materials are more accessible for the synthesis of RNA).

Anyway, the difference between the inside and outside of protocells is a relevant issue in the current context. Therefore, we addressed this issue by referring to this interesting paper.in the subsection “Some detailed mechanisms concerning the implementation of the model” of Methods (just after the explanation of *F_DO_*): “In fact, the introduction of this factor reflects our assumption about the difference between the interior and exterior of the protocells in the model system. Notably, a recent modeling study concerning RNA-based protocells suggested that the difference between the interior and exterior of the protocells may have been maintained owing to the intrinsic nature of the template-directed synthesis of RNA as a second-order autocatalytic process (i.e. both template and primer act as catalysts) [64].”.
