# Supplementary material for: From experimental clues to theoretical modeling: Evolution associated with the membrane-takeover at an early stage of life
Source: PLoS Comput Biol. 2025 Jun 13;21(6):e1012763. doi: 10.1371/journal.pcbi.1012763 (PMC12201655; doi:10.1371/journal.pcbi.1012763)
Supplement: Attachment — Submitted filename: Response to Reviewers-2.docx [file pcbi.1012763.s004.docx]

We thank the reviewers for their comments on our paper. The following is our response.

**Reviewer #1:**

The paper has been duly revised. ---Thanks.

*I think that there is a problem in the response to Reviewer 3. If growth of the surface is not proportional to the membrane surface are but loss from the membrane is, then indeed one should get a steady state.*

*Consider the equation*

*dA/dt = k - b A*

*where A is the surface. It is trivial that the equilibrium will be A = k/b.*

*If growth is also proportional to surface then we have*

*dA/dt = k A - b A = A(k-b),*

*and indeed if k>b then A growth exponentially.*

--- Yes, we agree to this point. To make it clearer, we have modified our relevant explanation (note the underlined words): “It should be noted that, for the sake of simplicity in the model, we assume that a protocell occupies a grid room. This may represent a limitation regarding the incorporation of amphiphiles into the membrane, since any protocell, regardless of size, is only surrounded by the four sides of its occupied grid room. In other words, it does not take into account the fact that “the larger the membrane surface area, the greater the chance of the membrane encountering surrounding amphiphiles that might join it” – in reality, a growing membrane may have a chance to grow faster. Fortunately, this simplification constitutes a conservative assumption for our topic here – that is, if a growing membrane has a chance to grow faster, the ribozyme favoring phospholipid synthesis would enjoy a greater selective advantage to spread by favoring the growth of protocells that contain it.”

**Reviewer #3:**

*The replies to the previous questions seem good. I have no further issues.* --- Thanks.
